# 

**Published:** 1896-08-15

**Authors:** 


					V
THE HOSPITAL. ABSOLUTELY PURE, therefore BEST, Auocst 15th, 1896,
CADBURY'S ww ^ CADBURY'S
attainable."?Lancct,
fHi:
cocoa ri\ Jfrll /M cocoa
" The standard of highest purity at present
Saturday,
August 15th, 1896.
> ^eidhiiiLtiAMa NShwich hospital . * ;
WITH EXTRA NURSING SUPPLEMENT. price twopence.
[Registered at tlie General Poet Offioe as a Newspaper for Monthh^Parts 6d ? fSlf'a i
transmistion iu tlio United Kingdom and Abroad.] S^r Annum^bV. SS^SSHtSt

				

